# Fluoroquinolone-Induced Sweet Syndrome: A Case Report

**DOI:** 10.7759/cureus.36952

**Published:** 2023-03-31

**Authors:** Aizaz R Chaudhry, Izza Iftikhar, Sameen A Choudhry, Rabia Islam, Hamza Islam

**Affiliations:** 1 Emergency Medicine, Bahawal Victoria Hospital, Bahawalpur, PAK; 2 Internal Medicine, Quaid-e-Azam Medical College, Lahore, PAK; 3 Emergency Medicine, Bahawal Victoria Hospital, Bahawalpur, PAK; 4 Research, Faisalabad Medical University, Faisalabad, PAK; 5 Research, Punjab Medical College, Faisalabad, PAK

**Keywords:** fluoroquinolone, erythema, neutrophilic infiltrate, non vascular, dermatosis

## Abstract

Sweet syndrome (SS) is a rare non-vasculitic neutrophilic dermatosis. Fever, the abrupt emergence of tender erythematous plaques and nodules, with an occasional presentation of vesicles and pustules along with dense neutrophilic infiltrates on skin biopsy are the hallmarks of the illness. Tender plaques or nodules develop along with other systemic manifestations suddenly in affected people which is considered to occur due to immune-mediated hypersensitivity.

We report a case of Sweet syndrome in Pakistan presenting in a 55-year-old female. It is worth reporting due to the rarity of such cases in this region. The patient was diagnosed after profound investigations and was treated with corticosteroid therapy.

## Introduction

Sweet syndrome (SS), also known as acute febrile neutrophilic dermatosis, is a rare non-vasculitic neutrophilic dermatosis [1]. Inflammatory skin lesions, fever, and blood neutrophilia are the hallmarks of SS [2]. Based on etiology, it is divided into three categories: malignancy-associated (most often leukemia but also breast or colon cancer), drug-induced (most commonly with granulocyte-colony stimulating factor), and classic SS. Phenotypic and histopathologic variants of SS may also be distinguished. Tender plaques or nodules that develop suddenly in affected people are often accompanied by fever, arthralgias, ophthalmologic symptoms, headaches, and in a very small percentage of cases, oral or genital lesions [3]. According to some experts, the mechanism of action involves immuno-mediated hypersensitivity or disruption of immunological function [4]. Diagnosis of SS is confirmed on skin biopsy. Regardless of the origin, systemic corticosteroids have a significant effect on the clinical and biochemical symptoms of Sweet syndrome. Although spontaneous remission is conceivable, the pattern has a persistent recurrent course over months [5].

## Case presentation

A 55-year-old female presented to the outpatient department with the complaint of a lesion on her left foot which was associated with swelling and she had this lesion for about a week. The patient also reported pain in the lesion. She had been suffering from fever and sore throat for two weeks as well. On exploration of drug history, she revealed that she was prescribed antibiotics for sore throat by the local healthcare unit. She was using ciprofloxacin. In combination with fluoroquinolone, she was using paracetamol for associated pyrexia. The skin lesion described started one day after starting the medication. Lesions aggravated progressively with the continuous intake of antibiotics according to the patient. And went on to spread to the contralateral lower limb and both upper limbs.

On further exploration of history, the patient narrated that those lesions which included plaques and nodules initially, progressed to arms and right leg within three days of starting the antibiotic regimen. Plaques started developing a bullous character. Tenderness was severe throughout the disease. With further progression of the disease, generalized swelling started on her right leg. This feature appeared on the third day after starting ciprofloxacin. The edema was pitting in nature. It extended from the knee joint to the toes. There was no history of trauma. History of any other organ system involvement was insignificant. The especially explored systems were the musculoskeletal and ocular systems. No remarkable finding was found in any other system. The patient had been taking antibiotics for seven days in total at the time of presentation to the dermatology clinic.

**Figure 1:**
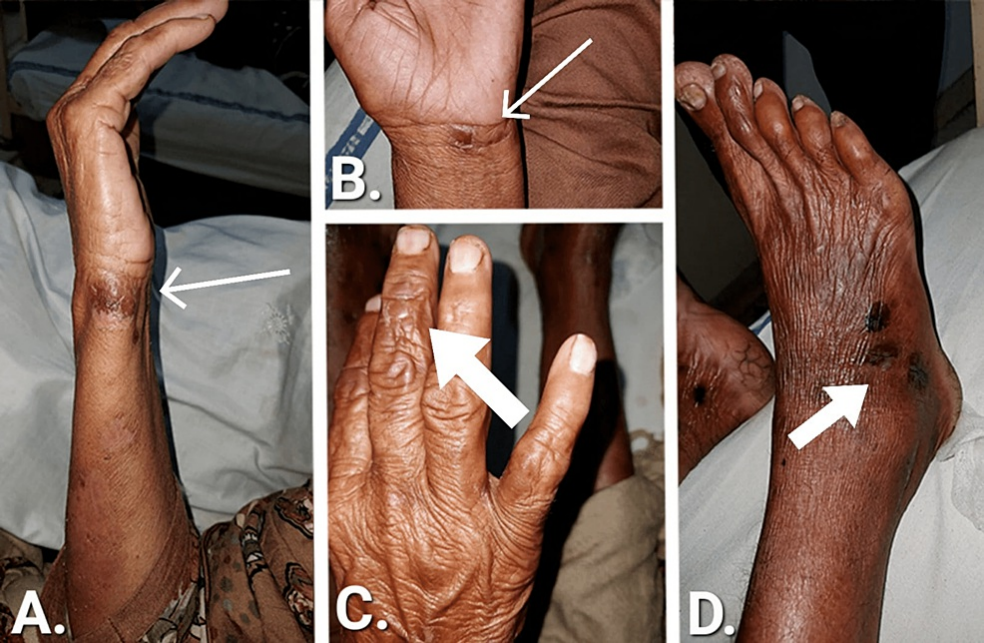
Lesions in different stages of drug-induced Sweet Syndrome A: Tender nodular lesion along the ulnar aspect of the right forearm; B: Newly forming tender nodule on the ventral aspect of the left hand; C: Bullous lesion on the middle finger of the right hand; D: Tender necrotic patches on the right foot

On detailed exploration of the lesions on the upper limb, there were multiple, discrete, erythematous plaques and nodules extending all over the limbs, especially on the dorsal aspect. Lesions were of different sizes and ranged from 1 cm2 to 2 cm2. A nodular lesion along the ulnar aspect of the right forearm, which was tender, is elaborated in Figure 1A. And newly forming tender nodules along the ventral aspect of the left hand are also highlighted in Figure 1B. These lesions progressed with swelling and tenderness observed on both hands. Moreover, the bullous lesion can be seen on the middle finger of the right hand in Figure 1C. There was mild to moderate pitting edema on the right leg along with tender necrotic patches on the right foot as highlighted in Figure 1D. As for the left leg, multiple bullous lesions on the dorsal surface with tense membrane and clear fluid were noted. Edema and necrotic areas were also added to the findings of the left leg.

For the initial investigations, complete blood count (CBC), liver function tests (LFTs), renal function tests (RFTs), serum electrolytes, and inflammatory markers were studied (Table 1). A general picture of acute infection was observed. The CBC acts as a screening test for underlying malignancy as well. As there were no features suggestive of anemia or thrombocytopenia, no further workup regarding malignancy was undertaken. Moreover, the absence of any suggestive feature of malignancy during history supported the decision.

**Table 1:** Clotting profile, CBC, LFTs, RFTs, serum electrolytes, inflammatory markers of the patient CBC: Complete blood count, LFT: Liver function test, RFT: Renal function test, INR: International normalized ratio, aPTT: Activated partial thromboplastin clotting time, HCT: Hematocrit, MCV: Mean corpuscular volume, MCH: Mean corpuscular hemoglobin, MCHC: Mean corpuscular hemoglobin concentration, ALT: Alanine transaminase, AST: Aspartate transaminase, ALP: Alkaline phosphatase, ESR: Erythrocyte sedimentation rate

Variable	Unit	Reference range
Coagulation profile		
Prothrombin time-control	11	10-14 seconds
Prothrombin time-patient	11	Up to 13 seconds
INR	1.0	0.9-1.3
Control time	28	25-35 seconds
aPTT	30	Up to 31 seconds
Hemogram		
WBC count	27.5	4-11 x 10^9^/L
Total RBC	4.3	3.8-5.2 x 10^12^/L
Hemoglobin	14.3	13-18 (g/dL)
HCT	40	35-46%
MCV	93.2	77-95 (fl)
MCH	28	26-32 (pg)
MCHC	33.1	32-36 (g/dL)
Platelets	231	150-400 x 10^9^/L
Neutrophils	85.9	40-80%
Lymphocytes	23	20-40%
Monocytes	5.1	2-10%
Eosinophils	3.3	1-6%
Renal function tests		
Urea (mg/dl)	40.55	10-50 mg/dl
Serum Creatinine (mg/dl)	0.7	0.5-0.9 mg/dl
Liver function tests		
Bilirubin total	0.3	0.3-1.2 mg/dl
Total protein	8.0	5.7-8.2 g/dl
Albumin	4.1	3.2-4.8 g/dl
ALT	46	Up to 40 U/L
AST	38	Up to 40 U/L
ALP	221	40-120 (U/L)
Serum electrolytes		
Sodium	133	135-145 mmol/L
Potassium	4.72	3.5-5 mmol/L
Chloride	98.8	98-107 mmol/L
Calcium	9.2	8.5-10.5 mg/dl
Inflammatory markers		
ESR	51	0-25 mm/1^st^ hour
C-reactive protein (quantitative)	126.1 mg/dl	<5

Among the differential diagnosis, erythema nodosum, deep venous thrombosis (DVT), and Sweet syndrome were considered. Doppler ultrasound of the left leg was performed. All the major vessels showed normal flow patterns. Saphenofemoral and saphenopopliteal junctions were competent as well. No evidence of DVT was found.

**Figure 2:**
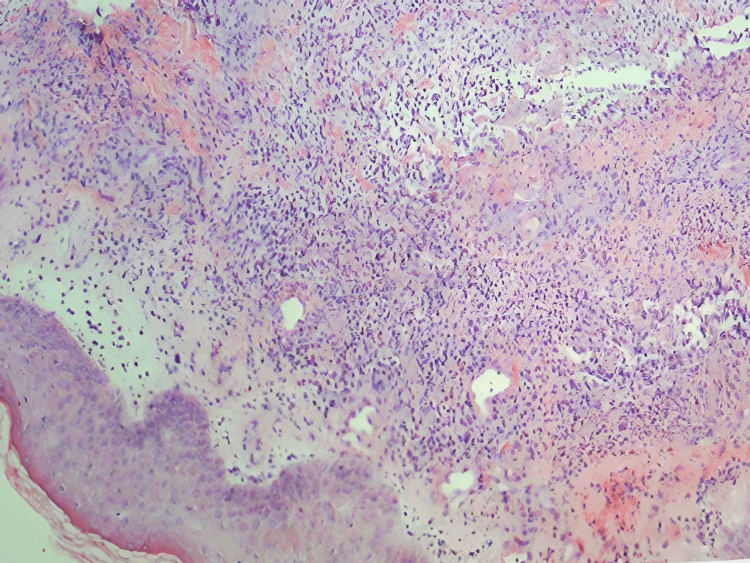
Hematoxylin-eosin staining of the skin biopsy reveal papillary edema and dermal infiltration with neutrophils

Initial signs and symptoms were favoring the differential diagnosis of erythema nodosum. However, the clinical picture of erythema nodosum and Sweet syndrome can overlap. Fortunately, a biopsy of the skin lesion is an essential prerequisite to establishing the final diagnosis in both cases. Hence a biopsy of the skin lesion was planned. To confirm the diagnosis, a punch biopsy was done which helped clear the clinical picture. The punch biopsy of a skin lesion showed dermal neutrophilic infiltration (Figure 2).

Laboratory investigation revealed leukocytosis (27,500/μl) with 85.9% neutrophils and an elevated erythrocyte sedimentation rate (ESR) (51 mm/1st hour) and C-reactive protein (CRP) (126.1 mg/l). Hence, based on the clinical picture, laboratory results, and skin lesion biopsy, a diagnosis of drug-induced Sweet syndrome was established. With the establishment of the diagnosis, the antibiotic was stopped. The treatment mainly consisted of corticosteroids which lessened the severity of symptoms with successive doses. 

## Discussion

Robert Douglas Sweet first described Sweet syndrome in 1964 which is also known as acute febrile neutrophilic dermatosis [6]. The symptoms of Sweet syndrome include fever, neutrophilia, erythematous plaques accompanying other lesions like papules on the skin, and the skin biopsy giving a picture of a dermal neutrophilic infiltration. Despite not being pruritic, these plaques are tender in nature [7]. There is no exact incidence rate regarding the epidemiology of drug-induced SS, especially due to ciprofloxacin. However, after careful research, the authors are convinced that there are fewer than 10 cases reported in the literature. In Pakistan, this is probably the first case being reported.

Our case clearly manifests Sweet syndrome. There are features of a classic febrile pattern, histopathologic evidence of a dense neutrophilic infiltrate, and abrupt onset of painful erythematous plaques or nodules. And most importantly there is a temporal relationship between drug ingestion and the onset of symptoms. Finally, the temporal relationship between the resolution of symptoms upon withdrawal of the drug and the start of steroid therapy was also documented. These features make the foundation for the diagnosis of Sweet syndrome as per the diagnostic criteria proposed by Walker and Cohen [8].

Sweet syndrome can present in different clinical settings ranging from classic (idiopathic), drug-induced, and malignancy-associated types. As there was no evidence of underlying malignancy in the given case, both classic and drug-induced Sweet syndrome were mainly under discussion. Features of classic and drug-induced Sweet syndrome overlap. But diagnostic criteria proposed by Walker and Cohen help in clearly distinguishing the two sister entities (Table 2) [8].

**Table 2:** Diagnostic criteria for classical Sweet syndrome versus drug-induced Sweet syndrome To establish a diagnosis of classic and malignancy-associated Sweet syndrome, two major criteria (1 and 2) and two of four minor criteria should be present (3, 4, 5, and 6) in a given case. To diagnose drug-induced Sweet syndrome all five criteria (A, B, C, D, and E) are required. ESR: Erythrocyte sedimentation rate, CRP: C-reactive protein

Diagnostic criteria for classical Sweet syndrome versus drug-induced Sweet syndrome	
Classical	Drug-induced
1. Abrupt onset of painful erythematous plaques or nodules	A. Abrupt onset of painful erythematous plaques or nodules
2. Histopathologic evidence of dense neutrophilic infiltrate without evidence of leukocytoclastic vasculitis	B. Histopathologic evidence of dense neutrophilic infiltrate without evidence of leukocytoclastic vasculitis
3. Pyrexia >38°C	C. Pyrexia >38°C
4. Association with an underlying hematologic or visceral malignancy, inflammatory disease, or pregnancy, OR preceded by an upper respiratory or gastrointestinal infection or vaccination	D. Temporal relationship between drug ingestion and clinical presentation, OR temporally-related recurrence after oral challenge
5. Excellent response to treatment with systemic corticosteroids or potassium iodide	F. Temporally-related resolution of lesions after drug withdrawal or treatment with systemic corticosteroids
6. Abnormal laboratory values at presentation (three of four): ESR >20 mm/hr; positive CRP; >8,000 leukocytes; >70% neutrophils	

In the case being discussed, a temporal association between ingestion of the drug and the onset of symptoms was present. Also, a close temporal association between the immediate resolution of symptoms and withdrawal of the drug was present. It was because of this pivotal feature that the classical type was ruled out and drug-induced Sweet syndrome was selected as the final diagnosis.

Sweet syndrome can also show extra-cutaneous symptoms in addition to skin and mucosal lesions. Internal Sweet syndrome manifestations in various organs such as the lungs, heart, pancreas, liver, kidneys, and central nervous system (CNS) have also been documented. Other signs of SS include involvement of the eyes, joints, and oral mucosa [9]. Pustules, vesicles, purpura, ulcers, and hemorrhagic lesions are a few more cutaneous manifestations that have been reported.

Usually, the presentation of features of Sweet syndrome is observed in the fourth to seventh decade of life. The incidence of the disease tends to peak in the spring and fall. Sweet syndrome has a female predilection with female to male ratio of 4:1 [10]. Our patient coincided with all the epidemiological and most of the clinical features reported in the literature.

Uncertainty surrounds Sweet syndrome pathophysiology. Cytokines deposition in the dermis is devised to be the pathological ground for the syndrome. Important cytokines in this regard may include interleukin (IL)-1, IL-6, IL-8, or granulocyte colony-stimulating factor (G-CSF). These may be responsible for the clinical features associated with Sweet syndrome [11].

The main treatment for Sweet syndrome is systemic corticosteroids (0.5 mg to 1 mg/kg/day for 4 to 6 weeks), which improve systemic symptoms and reduce skin lesions [12].

## Conclusions

Acute febrile neutrophilic dermatosis, also known as Sweet syndrome or Gomm-Button disease, is an uncommon form of inflammation that belongs to the group of neutrophilic dermatoses. Fever, abrupt emergence of uncomfortable erythematous plaques or nodules, and dense neutrophilic infiltrates on biopsy are the hallmarks of the illness. Classic, malignancy-associated, and drug-induced are the three subgroups of Sweet syndrome. Systemic corticosteroids are the mainstay treatment for SS.

This case report has the potential to be productive as the exact incidence, prevalence, and etiology of Sweet syndrome or any of its types, especially the drug-induced type is not clearly known. Fluoroquinolone-induced Sweet syndrome is rarely addressed in the literature. Sweet syndrome has a huge implication on the quality of life of patients because of its recurrent nature and disfiguring character. This case report is of great value as it uncovers an important side-effect of a very commonly used antibiotic. Moreover, this case report might prove useful for understanding pathogenesis, racial and gender predisposition, and the overall incidence of the disease.
